# Supplementary material for: Whole Blood Reactivity to Viral and Bacterial Pathogens after Non-Emergent Cardiac Surgery during the Acute and Convalescence Periods Demonstrates a Distinctive Profile of Cytokines Production Compared to the Preoperative Baseline in Cohort of 108 Patients, Suggesting Immunological Reprogramming during the 28 Days Traditionally Recognized as the Post-Surgical Recovery Period
Source: Biomedicines. 2023 Dec 21;12(1):28. doi: 10.3390/biomedicines12010028 (PMC10812925; doi:10.3390/biomedicines12010028)
Supplement: Supplementary file 1 [file biomedicines-12-00028-s001.zip › biomedicines-2651781-supplementary.pdf]

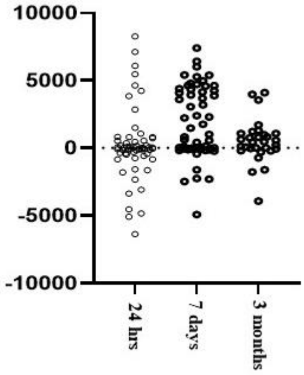


Supplementary Figure S1. Differences plot of IL-6 production after LPS stimulation compared to baseline. Significant heterogeneity was notable in response to LPS with some patients showing increase or decrease in IL-6 in response to LPS.
